# Including lifestyle medicine in undergraduate medical curricula

**DOI:** 10.3402/meo.v20.26150

**Published:** 2015-02-03

**Authors:** Edward Phillips, Rachele Pojednic, Rani Polak, Jennifer Bush, Jennifer Trilk

**Affiliations:** 1 Institute of Lifestyle Medicine, Joslin Diabetes Center, Harvard Medical School, Boston, MA, USA; 2 LevelSmart Consulting, Atlanta, GA, USA; 3 Department of Biomedical Sciences, University of South Carolina School of Medicine Greenville, Greenville, SC, USA

**Keywords:** behavior change, lifestyle medicine, physician education, undergraduate medical education, curriculum

## Abstract

**Purpose:**

Currently, there is no model to integrate the discipline of lifestyle medicine (LM) into undergraduate medical education. Furthermore, there are no guidelines, validated assessment tools, or evaluation or implementation plans in place.

**Background:**

The World Health Organization predicts that by 2020, two-thirds of disease worldwide will be the result of poor lifestyle choices. Fewer than 50% of US primary care physicians routinely provide specific guidance on nutrition, physical activity, or weight control.

**Methods:**

We are establishing a plan to integrate LM into medical school education in collaboration with the investing stakeholders, including medical school deans and students, medical curriculum developers and researchers, medical societies, governing bodies, and policy institutes. Three planning and strategy meetings are being held to address key areas of focus – with a particular interest in nutrition, physical activity, student self-care, and behavior change – to develop specific implementation guidelines and landmarks.

**Results:**

After the first two meetings, the proposed areas of focus were determined to be: 1) supporting of deans and key personnel, 2) creation of federal and state policy commitments, 3) use of assessment as a driver of LM, 4) provision of high-quality evidence-based curricular material on an easily navigated site, and 5) engaging student interest. Implementation strategies for each focus area will be addressed in an upcoming planning meeting in early 2015.

**Conclusion:**

This initiative is expected to have important public health implications by efficiently promoting the prevention and treatment of non-communicable chronic disease with a scalable and sustainable model to educate physicians in training and practice.

The World Health Organization predicts that by 2020, two-thirds of all disease worldwide will be the result of poor lifestyle choices (1). The public health and financial burden that accompany the millions of people with obesity and associated non-communicable chronic diseases continues to rise despite the strong scientific evidence supporting healthy behaviors (2) as an effective means of prevention and treatment. Although the most well-established chronic disease practice guidelines uniformly call for lifestyle change as first line therapy, fewer than 50% of primary care physicians routinely provide specific guidance on nutrition, physical activity, or weight control (3).

Lifestyle medicine (LM) curricula reform in undergraduate medical education (UME) is a logical, critical, and strategic step to alter the preventive care landscape (4). The lack of training regarding physical activity was recognized in a 1975 survey that revealed only 16% of medical schools offered curriculum geared toward exercise (5). A similar survey in 1985 found that only 20% of medical schools had a required nutrition course (6). In 2014, thought leaders in nutrition made a call for action after just 27% of medical schools indicated that they provided the 25 h of recommended nutrition education, with most averaging only 19.6 h (7). Furthermore, although 61% of medical school leaders reported that it was the responsibility of medical schools to educate students about physical activity, only 6% reported having a core course or required curriculum that addresses exercise prescription (8).

In order for health care to transcend the looming public health and financial burden, physicians must become experts in the fundamentals of LM, defined in the *Journal of American Medical Association* as the ‘evidence-based practice of assisting individuals and families to adopt and sustain behaviors that can improve health and quality of life’^4^. Medical students themselves recognize the need for a formalized curriculum in LM as well as the lack of training they currently receive. Although 94% of US medical students perceived the competence to prescribe a physical activity as either ‘moderately important’ or ‘important’ (9), only 10% of graduates felt capable of doing so (2). Moreover, in another survey of medical students, 72% of freshmen students judged nutritional counseling as highly relevant, but this sentiment declined to 46% by their senior year (10). Training medical students in LM throughout all 4 years of UME will create a new generation of physicians who have the knowledge, skills, and tools to improve and sustain their own health behaviors and that of their patients.

In September 2013, led by the Institute of Lifestyle Medicine, Joslin Diabetes Center, Harvard Medical School, the Josiah Macy Jr. Foundation sponsored a 2-day LM think tank at the University of South Carolina School of Medicine, Greenville, SC, to kick-off an effort to transform medical education. A second meeting, sponsored by the Ardmore Institute of Health, was held in August 2014 in Boston, MA, to establish key tactics and strategies for implementation. Participants, including medical school deans, medical students, content experts, and representatives of professional associations, government agencies, accreditation agencies, and national assessment boards, engaged in a broad and extensive discussion. In this short communication, we provide a summary of the discussions and recommendations that resulted from the initial two meetings.

These meetings were the first in which thought leaders in LM had the opportunity to discuss actionable strategies to equip future physicians to practice LM. Carefully considering the previously outlined definition of LM and core competencies for physicians (4), the committee worked systematically through identifying 1) the key stakeholders, 2) the principle areas of focus for curricula, and 3) the next steps for integrating LM into medical schools. The product of these two meetings was a developed vision statement and five focus areas from which to further develop working groups, strategies, and tactics to move the LM initiative forward in UME. This collaborative is an essential first step to establishing a long-term implementation plan for integrating LM into medical school education.

## Vision statement

The participants of the think tank defined the goal of integrating LM into medical education with the following vision statement, ‘Our vision is to integrate lifestyle medicine into medical education. Lifestyle factors including nutrition, physical activity, and stress are critical determinants of health, causing a pandemic of chronic disease and unsustainable health care costs. We will provide an array of evidence-based curricular resources for prevention and treatment of lifestyle related diseases throughout medical education’.

## Principle areas of focus for LM curricula

Participants acknowledged that LM has multiple components and principle areas of initial focus were narrowed to: 1) physical activity, 2) nutrition, 3) medical student's self-care, and 4) behavioral change.

Topics less mature in their evidence base (e.g., stress management) or already widely taught in UME (e.g., tobacco, alcohol, and other substance abuse) were not included. Inclusion of a medical student/physician health model was deemed essential to emphasize the significant impact of a healthier student/physician and translation toward improving health behaviors of patients.

Participants also determined that a large credible body of LM curricula material currently exists and efforts would best be focused on leveraging available resources to improve adoption rather than drafting new curricula. Finally, medical students are learning in a much more technologically advanced and rapidly changing environment than historical medical education settings, and these realities must be met by future curriculum designs for successful implementation.

## Determining essential stakeholders and infrastructure

Opportunities and challenges to implementing LM curricula were focused on identifying necessary infrastructure and key stakeholders and determining the needs of those constituency groups. Strategies to support the LM curriculum implementation goals were determined as follows:

1. Support of deans. The support of medical school deans, particularly curricular deans, is seen as an essential component in the integration of LM. As such, LM curriculum will be made available to medical school deans and the collaborative will work with the curricular staff to integrate LM as it works best with the current fixed curriculum. The LM initiative will not expect ‘mandates’ to schools or deans and is intended to be integrated as appropriate by each medical school.
2. Student interests. Student interest groups and overall engagement is critical for the acceptance and dissemination of LM curricula. By advocacy and participation in peer-led interest groups, medical students will be able to network with curricular staff, clinicians, and researchers to become informed and engaged in the timely best practices of LM.
3. Assessment as a driver of LM. Currently, test items on the national medical boards do not address LM directly. Questions will need be identified, modified, and added by the National Medical Board of Examiners to represent the importance of LM in medical practice. However, it is critical that the assessment of knowledge and skills of LM competencies is implemented and received as a promoter of beneficial skills.
4. Evidence-based medicine. Emerging literature has demonstrated an evidence-based line of support for the implementation of LM in practice. A web-based platform is being developed to house readily available evidence-based resources for curriculum development, and must be expanded and updated to provide support for the implementation of LM in practice.
5. Congressional and state policy/support. With the implementation of the Affordable Care Act as well as a shifting landscape of health care reimbursement, state and federal support is required for impactful and lasting change within the delivery of medical care. A constituent group consisting of the LM think tank, the Bipartisan Policy Center, and the American College of Sports Medicine has been formed to open communication, inform local- and national-elected officials, and address potential necessary policy challenges.

In order to continue progress on this initiative, a subsequent grant from the Ardmore Institute of Health will support two planning meetings for 2014 and 2015. Working groups for each of the five focus areas are being established and strategies are being developed with key personnel to integrate LM into UME.

## Conclusion

To effectively address the root cause of the majority of health care costs, prevalence of noncommunicable chronic diseases, and causes of death (1, 2), it is imperative that LM competencies are integrated now into the education of medical students throughout their training. The impetus for reforming medical education to address preventable causes of chronic disease is bolstered by several significant policy initiatives (11), including The Affordable Care Act, which requires health insurers to cover recommended preventive services (12), and The United States National Physical Activity Plan©, which advocates the promotion of physical activity education in the training of all health care professionals (13). Across party lines this need is being recognized as the Bipartisan Policy Center has issued two timely reports 1) *Lots to Lose: How America's Health and Obesity Crisis Threatens our Economic Future* (14), which proposes that ‘nutrition and physical activity training should be incorporated in all phases of medical education: medical schools, residency programs, credentialing processes, and continuing education requirements’; and 2) *Teaching Nutrition and Physical Activity in Medical School: Training Doctors for Prevention-Oriented Care* (15), which calls directly to ‘develop and implement a standard nutrition and physical activity curriculum’ for medical students and is listed among ‘… action items where substantial progress is possible in the next year’.

To accomplish this essential and timely task, experts across the nation are being assembled who are committed to incorporating LM into medical education in the United States. Many partnerships have been forged and the strength of many will facilitate this essential endeavor, which is vital for transforming the US health care system. As the LM initiative gains momentum, further research is needed to determine the most effective method to showcase LM resources, integrate LM content into standardized undergraduate curricula, and assess LM knowledge and competencies.
